# Designing a magnesium alloy with high strength and high formability

**DOI:** 10.1038/s41467-018-04981-4

**Published:** 2018-06-28

**Authors:** T. T. T. Trang, J. H. Zhang, J. H. Kim, A. Zargaran, J. H. Hwang, B.-C. Suh, N. J. Kim

**Affiliations:** 1 Graduate Institute of Ferrous Technology, Pohang University of Science and Technology, Pohang, 37673 Republic of Korea; 2 College of Material Science and Chemical Engineering, Harbin Engineering University, Harbin, 150001 China; 3 Korea Institute of Materials Science, Changwon, 51508 Republic of Korea

## Abstract

Although magnesium alloys, as the lightest structural alloys, offer significant potential for automotive applications, their applications remain limited due to their poor formability at room temperature. Since the strategies used for improving formability usually result in a degradation of strength, there are no high strength magnesium alloys showing good formability. Here we report an alloy design concept that can simultaneously provide high strength and good formability. Such designed alloy when subjected to an appropriate processing technique shows a combination of strength and formability that surpasses those of the existing magnesium alloys reported so far. The alloy design concept used in the present study is based on the utilization of alloying elements that can induce precipitation, as well as maximize the segregation of other texture-controlling alloying elements. Such developed alloy is expected to broaden the application of Mg alloy sheets, which are now starting to gain acceptance by automotive industries.

## Abstract (web summary)

Despite being the lightest structural alloys, obtaining magnesium alloys with both high strength and high formability remains a challenge. Here, the authors use precipitation and elemental segregation to design a magnesium alloy with high strength and high formability.

## Introduction

**Fig. 1:**
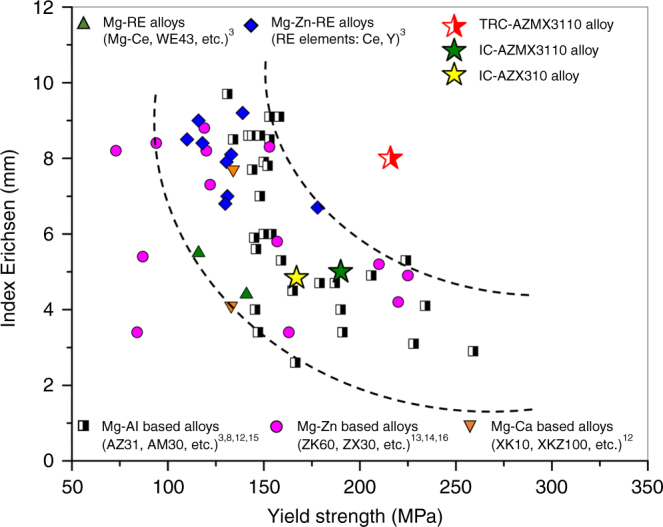
Yield strength and stretch formability represented by the Index Erichsen (IE) value at room temperature of various Mg alloys sheets. Higher IE values mean that the alloys exhibit better formability. Overall, Mg alloys display an inverse relationship between IE value and yield strength, as illustrated by the area between two dash lines. The developed TRC-AZMX3110 alloy shows a much better combination of strength and formability than others. Different Mg alloy systems are marked by different colors and symbols and the corresponding references are cited

Magnesium alloys, as the lightest structural alloys having a density about one-fourth that of steels, offer significant potential for improving energy efficiency of various transportation systems such as automobiles^1,2^. Numerous R&D efforts in the last decade have led to the development of various types of magnesium alloys having good combination of strength and ductility^3,4^ and excellent corrosion resistance^5^. Although there are many variants of magnesium alloys showing large ductility, however, they usually show poor formability at room temperature^3^, hindering their widespread applications in automobiles. Poor formability of magnesium alloys arises from several factors; development of strong basal texture during rolling (thermomechanical treatment) and limited number of available deformation modes due to magnesium having hexagonal close-packed (hcp) structure^6^. Although Mg alloys are readily formable at warm or high temperatures despite their poor formability at room temperature, forming at warm or high temperatures is quite energy intensive and inefficient. Therefore, to make Mg alloys attractive for applications in automobiles, their formability at room temperature should be improved. There have been numerous attempts to improve formability of magnesium alloys and it has been shown that the formability of Mg alloys can be improved by texture randomization/weakening from the typical strong basal texture of conventional Mg alloys, which is usually achieved by the modification of thermomechanical processing^7,8^ and the addition of rare earth elements (REEs)^9–11^. The randomized/weakened basal texture of REE-containing alloys is certainly beneficial for formability, but also results in low strength. Such inverse relationship between formability and strength is also applicable to other non-REE containing Mg alloys^3^. Figure 1 shows the Index Erichsen (IE) value (which is an indication of stretch formability; see Methods) as a function of yield strength of various Mg alloys^3,8,12–16^. As shown in Fig. 1, yield strength of Mg alloys having the IE values larger than 8 mm is lower than ~160 MPa. However, as yield strength of Mg alloys increases to 200 MPa, their IE values become lower than 6 mm, making them non-formable at room temperature. It is apparent that the conventional approaches that improve formability have adverse effects on strength or vice versa. Therefore, new alloy design and processing concepts should be utilized to develop high strength Mg alloys with good formability at room temperature.

In this study, we report an alloy design concept that can simultaneously provide high strength and good formability at room temperature. The newly developed Mg-3Al-1Zn-1Mn-0.5Ca (wt.%) (AZMX3110) alloy using the alloy design concept shows a much improved combination of formability (IE value) and yield strength, namely IE value of 8 mm with yield strength of 219 MPa, as compared to commercial as well as experimental Mg alloys, as presented in Fig. 1. Such improvement in the combination of formability and strength has been realized by a judicious control of alloy chemistry and a selection of appropriate process for such alloy chemistry. The composition of AZMX3110 alloy is quite similar to that of the most familiar and commonly used Mg-3Al-1Zn (AZ31) alloy. It is expected that such composition of the developed alloy would lead to an early acceptance by the users due to its similarity to AZ31 alloy.

## Results and discussion

### Alloy design concept and selection of fabrication processes

AZMX3110 alloy was designed to utilize a beneficial effect of Zn/Ca co-segregation along grain boundaries on randomizing/weakening texture^17^ and to promote precipitation of intermetallic compounds that can increase strength. One important aspect to consider when choosing the alloy chemistry was that the alloying elements responsible for precipitation should not interfere with segregation of Zn and Ca along grain boundaries. As mentioned previously, Mg-Zn-Ca (ZX) based alloys usually show good formability^18–20^, but increasing Zn content does not lead to an increase in strength in as-annealed condition^18,20^, which gives the maximum formability. On the other hand, when Al, one of the most common alloying elements, is added to Mg, there is an increase in strength with an increase in Al content^15,21^. However, when Al is added to ZX alloys, the formation of Al_2_Ca particles becomes inevitable, which might reduce the degree of Ca segregation along grain boundaries resulting in a less desirable basal texture. Therefore, to induce the precipitation of intermetallic compounds while simultaneously providing segregation of Zn and Ca along grain boundaries, the co-additions of Al and Mn were considered. It is expected that the co-additions of Al and Mn to a ZX alloy would result in precipitations of Al_8_Mn_5_ and Al_2_Ca in the microstructure based on thermodynamic calculation. However, since Al_8_Mn_5_ is the primary phase forming in the melt, its formation would reduce the amount of Al that can react with Ca, thereby reducing the amount of Al_2_Ca and concurrently freeing most of Ca, which can be segregated along grain boundaries. The composition of Mg-3Al-1Zn-1Mn-0.5Ca in wt.% (AZMX3110) was selected, which can be regarded as a variant of the most familiar and commonly used Mg-3Al-1Zn (AZ31) alloy.

The AZMX3110 alloy was fabricated using twin-roll casting (TRC), which is known for its cost effectiveness and ability to produce high quality Mg alloy sheets^13,22,23^. TRC process is ideal for the fabrication of the AZMX3110 alloy since the alloy involves the formation of Al_8_Mn_5_ and Al_2_Ca particles at high temperatures and they can become quite coarse if the alloy is solidified with a slow solidification rate such as during conventional ingot casting (IC). TRC process, on the other hand, provides a high solidification rate, resulting in a refinement of constituent phases^23^. To show the effectiveness of the TRC process in fabricating AZMX3110 alloy, the alloy with the same composition was fabricated by conventional IC. In addition, to validate the alloy design concept utilized in the present study, AZX310 alloy without Mn was also fabricated by IC. The microstructure and mechanical properties of these two IC alloys are compared with those of TRC-AZMX3110 alloy. The analyzed compositions of the alloys are shown in Supplementary Table 1. In addition, their tensile stress-strain curves and tensile properties are shown in Supplementary Fig. 1 and Supplementary Table 2, respectively.

### Microstructure

**Fig. 2:**
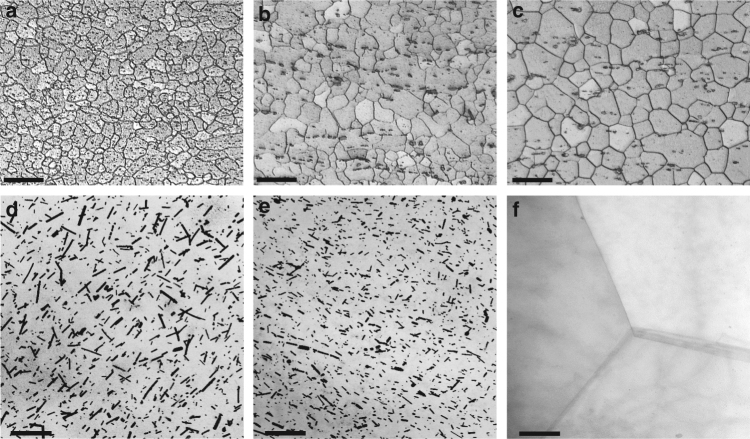
Microstructure of the studied Mg alloys. **a–c** OM images of TRC-AZMX3110 (**a**), IC-AZMX3110 (**b**), and IC-AZX310 (**c**) showing grain size and distribution of coarse second phases particles. Scale bar of OM images corresponds to 20 μm. **d–f** STEM images of TRC-AZMX3110 (**d**), IC-AZMX3110 (**e**), and IC-AZX310 (**f**) showing the presence of fine plate-shaped particles in TRC-AZMX3110 and IC-AZMX3110 and the absence of such fine particles in IC-AZX310. Scale bar of STEM images corresponds to 500 nm

Figure 2 shows the typical microstructure of TRC-AZMX3110 alloy, along with those of IC-AZMX3110 and IC-AZX310 alloys. The TRC-AZMX3110 alloy (Fig. 2a) shows a homogenous distribution of fine (~5 μm) equiaxed grains, while IC-AZMX3110 alloy (Fig. 2b) shows a rather inhomogeneous microstructure containing both coarse and fine grains with an average grain size of 8 μm. As compared to both TRC- and IC-AZMX3110 alloys, IC-AZX310 alloy shows somewhat coarser microstructure with an average grain size of 11 μm (Fig. 2c). Besides such difference in grain size, there are differences in the second phase particles present in the microstructure. The fraction of coarse second phase particles observable in optical micrograph is higher in IC-AZMX3110 alloy than in IC-AZX310 and TRC-AZMX3110 alloys (area fraction: 3.6, 2.0, and 1.9 %, respectively). Also, the size of second phase particles in TRC-AZMX3110 alloy is much finer (average size: ~500 nm) than those in IC-AZMX3110 (average size: ~1.5 µm) and IC-AZX310 (average size: ~1.2 µm) alloys. The above results indicate the beneficial effect of TRC on the refinement of microstructural constituents as reported previously for various Mg alloys^13,22,23^. EPMA (Supplementary Fig. 2) and TEM (Supplementary Fig. 3) analyses show that while all the particles in IC-AZX310 alloy are Al_2_Ca, IC- and TRC-AZMX3110 alloys contain both Al_2_Ca and Al_8_Mn_5_ particles, as expected.

In addition to coarse second phase particles, there are many fine particles within grains of both TRC- and IC-AZMX3100 alloys (Fig. 2d, e, respectively), while there are virtually no particles within grains of IC-AZX310 alloy (Fig. 2f). The particles have plate shape and are randomly oriented within matrix. The particles in TRC-AZMX3110 alloy are longer (average length of 66 nm, average width of 17 nm, aspect ratio of 3.9) than those in IC-AZMX3110 alloy (average length of 31 nm, average width of 15 nm, aspect ratio of 2.1). They are mostly Al_8_Mn_5_ particles as identified by TEM analyses (Supplementary Fig. 4a, b, and c). In addition, there is also a small amount of Al_8_Mn_4_Ca particles (Supplementary Fig. 4d, e and f). There are no fine Al_2_Ca particles in the microstructure of both TRC-AZMX3110 and IC-AZMX3110 alloys.

### Grain boundary segregation

**Fig. 3:**
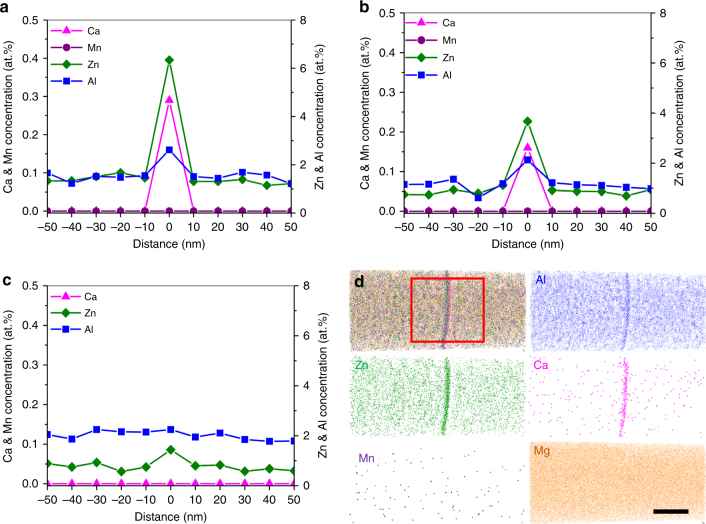
Segregation of solute elements at grain boundaries. **a–c** TEM-EDS line scans across grain boundaries of TRC-AZMX3110 (**a**), IC-AZMX3110 (**b**), and IC-AZX310 (**c**). There are significant amounts of Ca, Zn and Al segregated along grain boundaries of TRC-AZMX3110 alloy, whereas smaller amounts of these solute elements are segregated along grain boundaries of IC-AZMX3110 and IC-AZX310. **d** Atom Probe Tomography (APT) reconstruction of 45 × 45 × 100 nm^3^ volume containing a grain boundary in TRC-AZMX3110. The enrichments of Ca, Zn, and Al along grain boundary are clearly demonstrated (marked by red square). Scale bar corresponds to 20 nm

Energy dispersive X-ray spectroscopy (EDS) analysis was conducted to study the segregation behavior of alloying elements along grain boundaries. There are considerable amounts of Zn and Ca as well as Al segregated along grain boundaries of TRC-AZMX3110 alloy (Fig. 3a). On the other hand, both IC-AZMX3110 and IC-AZX310 alloys show smaller amounts of Zn and Ca segregated along grain boundaries than TRC-AZMX3110 alloy (Fig. 3b, c). Grain boundary of TRC-AZMX3110 alloy was also subjected to the atom probe tomography (APT) analysis. The APT construction in Fig. 3d shows that grain boundary (marked by red square) of TRC-AZMX3110 alloy is enriched with Al, Zn, and Ca, confirming the results of EDS analysis. A segregation of Mn along grain boundaries does not occur, which is in contrast to an observation of Somekawa et al. showing Mn segregation in a binary Mg-0.65Mn (wt.%) alloy^24^. An absence of Mn along grain boundaries of the present AZMX3110 alloy might be because Mn reacts with Al to form primary Al_8_Mn_5_ particles within Mg melt and plate-shaped Al_8_Mn_5_ particles within Mg matrix so that there is virtually no Mn left within solid solution. On the other hand, no such reaction occurs in a binary Mg-0.65Mn (wt.%) alloy. Although Mn does not segregate along grain boundaries of the present AZMX3110 alloy, Mn promotes, albeit indirectly, the segregation of Zn and Ca along grain boundaries. As mentioned previously, there is a formation of Al_2_Ca in the present alloy, which reduces the amount of Ca that can be segregated along grain boundaries. An addition of Mn results in the precipitation of Al_8_Mn_5_ as the primary phase in the melt and reduces the amount of Al that can react with Ca, thereby reducing the amount of Al_2_Ca and concurrently freeing most of Ca, which can be segregated along grain boundaries. The presently observed considerable segregation of Zn and Ca along grain boundaries has also been reported in Mg-0.3Zn-0.1Ca (at. %) alloy^17^. The main driving force for the co-segregation of Zn and Ca atoms along grain boundaries is known to be the minimization of elastic strains of the dislocations in grain boundaries induced by size misfit between solute atoms (Zn and Ca) and Mg. Zn atom with smaller size than Mg atom tends to segregate to the compression region of dislocation core in grain boundaries, whereas Ca atom with larger size than Mg atom tends to segregate to the extension region. Such co-segregation of Zn and Ca is reported to result in greater reduction of grain boundary energy than the individual segregation of Zn or Ca atoms^17^. In addition, a mixing enthalpy between Zn and Ca is negatively much larger than the Mg-Zn and Mg-Ca combinations, indicating a strong attraction interaction between Zn and Ca atoms^25^. Both of these would contribute to an increased tendency for Zn and Ca atoms to co-segregate along grain boundaries.

### Texture

**Fig. 4:**
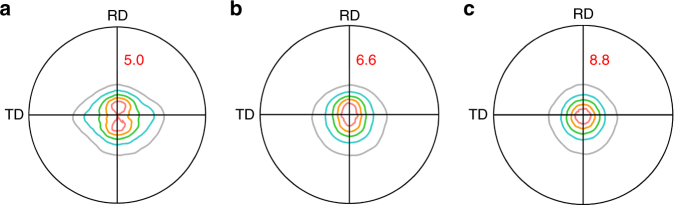
Texture of the studied Mg alloys. Pole figures of basal (00.1) planes showing the texture of TRC-AZMX3110 (**a**), IC-AZMX3110 (**b**), and IC-AZX310 (**c**). The numbers in red color indicate the maximum texture intensity. While IC-AZMX3110 and IC-AZX310 exhibit a typical basal texture, the texture of TRC-AZMX3110 is characterized by a broadening of basal poles along the transverse direction (TD) and a splitting of basal poles toward the rolling direction (RD) from the normal direction (ND) of the sheet. In addition, the maximum texture intensity of TRC-AZMX3110 is lower than those of IC-AZMX3110 and IC-AZX310

As shown in Fig. 4, there is a noticeable difference in the texture between TRC-AZMX3110 alloy and IC-AZMX3110 and IC-AZX310 alloys. TRC-AZMX3110 alloy shows a texture having a broadening of basal poles along the transverse direction (TD) with a splitting of basal poles along the rolling direction (RD) (Fig. 4a), similar to those of AT31 alloy^22^ and Zn and Ca/Y containing low-solute alloys^12,26,27^. On the other hand, IC-AZMX3110 and IC-AZX310 alloys show a typical basal texture (Fig. 4b, c). In addition, the maximum intensity of basal poles is lower in TRC-AZMX3110 alloy than in IC-AZMX3110 and IC-AZX310 alloys (5.0 vs. 6.6 and 8.8). Such difference in the texture among the alloys is mainly due to the above-mentioned differences in the degree of segregation of Zn and Ca along grain boundaries among three different alloys. It has been reported that the co-segregation of Zn and Ca decreases grain boundary energy and induces grain boundary pinning effect during recrystallization and grain growth, thereby reducing the grain boundary mobility and inhibiting grain growth into preferable orientation (i.e., basal texture orientation)^17^. In the case of IC-AZMX3110 and IC-AZX310 alloys, on the other hand, they show typical strong basal texture after annealing due to a much less degree of segregation of Zn and Ca along grain boundaries. As can be seen here, the quadruple nature of the texture of TRC-AZMX3110 alloy is much more desirable for formability than the strong basal texture of IC-AZMX3110 and IC-AZX310 alloys. Although the texture of TRC-AZMX3110 alloy is not perfectly random, it is the second best texture since the broadened angular distribution of basal poles in one direction (transverse direction in the present case) and the split basal poles with maximum intensity in the orthogonal direction (rolling direction in the present case) provide more uniform deformation during bi-axial stretch forming than the texture with distribution of basal poles mostly along one direction such as in REE-containing alloys, and of course the basal texture^3,10,11^.

### Room temperature formability and tensile properties

As a result of texture modification, TRC-AZMX3110 alloy shows high formability at room temperature, with the IE value of 8.0 mm. On the other hand, IC-AZMX3110 and IC-AZX310 alloys having typical basal texture show low IE values of 5.0 mm and 4.8 mm, respectively, which are similar to those of previously developed alloys as shown in Fig. 1. An additional important characteristic of TRC-AZMX3110 alloy is that it shows such high formability at high yield strength of 219 MPa, which is higher than those of IC-AZMX3110 (190 MPa) and IC-AZX310 (167 MPa) alloys. Such high strength of TRC-AZMX3110 alloy is mainly due to its fine grain size as well as the presence of fine Al_8_Mn_5_ particles within matrix. As shown in Fig. 2a–c, TRC-AZMX3110 alloy shows a finer grain size (5 μm) than IC-AZMX3110 (8 μm) and IC-AZX310 (11 μm) alloys. Using the published data^28^ on the strengthening coefficient (*k*_*y*_) of 209-319 MPa•μm^1/2^, it can be seen that the yield strength of TRC-AZMX3110 alloy would be higher by 19-30 MPa and 30-47 MPa as compared to those of IC-AZMX3110 and IC-AZX310 alloys, respectively, by grain refinement. Regarding the precipitation strengthening, the most important variables for plate shape particles are the planar inter-particle spacing and planar diameter of particles^29–31^. Measurement of the inter-particle spacing by a linear intercept method shows that TRC-AZMX3110 alloy has only slightly larger inter-particle spacing than IC-AZMX3110 alloy (151 nm vs. 141 nm). Using the formula, *d*_*p*_ = (*L•W*)^*1/2*^ (where *d*_*p*_ is the planar diameter, and *L* and *W* are length and width of plate shape particles, respectively)^31,32^, the planar diameter of particles was calculated to be 33.5 nm and 21.6 nm for TRC- and IC-AZMX3110 alloys, respectively. Using these values to calculate the increment in strength by Orowan strengthening mechanism assuming the operation of basal slip with Schmid factor of 0.3^33^, it has been shown that the contribution of precipitation strengthening is almost same for TRC- (106 MPa) and IC-AZMX3110 (103 MPa) alloys. It should be noted that the amount of precipitation strengthening cannot be reliably estimated by using the equations on precipitation strengthening of Mg alloys^29–32,34^, since the particles in both TRC- and IC-AZMX3110 alloys are randomly oriented. Although the calculated contributions of grain size and fine particles to overall strength appear to be overestimated when one compares the strength of IC-AZX310 alloy with those of other alloys, it is clear that the fine grain size and fine particles in TRC-AZMX3110 alloy are responsible for its high strength.

Other low-Al-containing alloys such as Mg-1Al-1Zn-0.1Ca-0.5Mn (wt.%) alloy^26^ and Mg-1.1Al-0.3Ca-0.2Mn-0.3Zn (at.%) alloy^12^ also show a quadruple texture and good formability (IE value: 7.6–7.7 mm), however, an absence of strengthening precipitates due to their low Al contents gives lower yield strength (<150 MPa) than the present TRC-AZMX3110 alloy. It should be noted that these low-solute alloys would show good formability even when subjected to IC because they have only a small amount of Al and accordingly small amount of Al_2_Ca particles so that most of Ca and Zn could segregate along grain boundaries, although the segregation behavior of Ca and Zn along grain boundaries was not reported in these alloys^12,26^. On the other hand, high-solute AZMX3110 alloy shows good formability when subjected to TRC, but not when subjected to IC since there is not much Ca segregated along grain boundaries due to a formation of Al_2_Ca during IC.

In summary, our alloy design concept combined with appropriate processing technique allows for the development of a Mg alloy having an excellent combination of mutually exclusive properties, high strength and high formability at room temperature, which surpasses those of other commercial and experimental Mg alloys. Furthermore, the composition of the developed alloy is quite similar to that of the most familiar and commonly used AZ31 Mg alloy so that the developed alloy can be expected to gain an early acceptance by users such as automotive manufacturers.

## Methods

### Fabrication and thermomechanical treatment process

TRC-AZMX3110 alloy was produced by twin-roll casting (TRC) process. The alloy was melted under an inert atmosphere of CO_2_ and SF_6_ mixture and the molten alloy was transferred into preheated tundish, the temperature of which was set at 750 °C, followed by TRC. The roll gap was set at 1.8 mm and roll speed was 4 m per minute, similar to the processing conditions used for fabricating other Mg alloys^23^. To compare the effects of casting methods and alloying elements on microstructure and mechanical properties, additional AZMX3110 and AZX310 alloys were produced by ingot casting (IC) process, namely IC-AZMX3110 and IC-AZX310 alloys, respectively. The IC alloys were produced by induction melting at 900 °C in a graphite crucible under an inert atmosphere of CO_2_ and SF_6_ mixture and were cast into a copper mold. Analyzed chemical compositions of the alloys are shown in Supplementary Table 1. The alloys were subsequently homogenized at 450 °C for 12 h, followed by water quenching. After homogenization, TRC-AZMX3110 alloy was hot rolled at 300 °C, and IC-AZMX3110 and IC-AZX310 alloys were hot rolled at 300 and 400 °C to a final thickness of 1.2 mm. However, the sheets of IC-AZMX3110 and IC-AZX310 alloys were fractured during rolling at 300 °C, and therefore only the sheets of IC-AZMX3110 and IC-AZX310 alloys rolled at 400 °C were subjected to further analyses. After rolling, the alloys were given final annealing at 350 °C for 1 h, followed by water quenching. Thermodynamic calculations of the phase evolution were conducted by FactSage software version 7.1.

### Tensile test and Erichsen test

Longitudinal tensile tests were conducted using the specimens with a gauge length of 12.5 mm, a gauge width of 5 mm, and a gauge thickness of 1 mm at a strain rate of 6.4 × 10^−4^ s^−1^. Stretch formability was evaluated by the Erichsen test (disc shape specimen with 50 mm diameter). The punch diameter and speed used were 20 mm and 0.1 mm s^−1^, respectively. A blank holder force was 10 kN and silicon oil was used as a lubricant.

### Sample preparation for microstructure analyses

Microstructural features were characterized using optical microscopy (OM), electron probe microanalyzer (EPMA), transmission electron microscopy (TEM) coupled with energy dispersive X-ray spectroscopy (EDS) and atom probe tomography (APT). TEM specimens were mechanically grinded to a thickness of 100 nm, then were punched into 3 mm diameter discs. These discs were electro-polished in a solution of 60 vol.% methanol, 30 vol.% glycerol, and 10 vol.% nitric acid at −5 °C, using a twin-jet polisher, followed by ion milling. TEM image observations were conducted on a JEOL JEM-2100F at an accelerating voltage of 200 kV and EDS analyses were conducted on a JEOL JEM-2100F equipped with double spherical aberration correctors at an accelerating voltage of 200 kV. Atom probe tomography (APT) specimen was fabricated using dual-beam FIB. APT experiments were performed on a Cameca LEAP4000X HR under ultraviolet laser pulse mode at a pulse energy of 50 pJ and a pulse rate of 125 Hz. ATP data reconstructions were performed using IVAS3.8 software.

### Texture analyses

The crystallographic texture of the alloys was analyzed on the midsection of the specimens using Mo K_α_ radiation at 3 kW. Pole figures were obtained from five different planes, (10.0), (00.2), (10.1), (10.2), and (10.3), using the Schulz reflection method, and the texture data were analyzed using TexTool v. 3.3 software.

### Data availability

All data are available from the corresponding authors on reasonable request.

## Electronic supplementary material

Supplementary Information
