# Supplementary material for: Designing a magnesium alloy with high strength and high formability
Source: Nat Commun. 2018 Jun 28;9:2522. doi: 10.1038/s41467-018-04981-4 (PMC6023917; doi:10.1038/s41467-018-04981-4)
Supplement: Supplementary file 1 — Supplementary Information [file 41467_2018_4981_MOESM1_ESM.pdf]

Supplementary data

Designing a Magnesium Alloy with High Strength and High Formability

Trang et al.

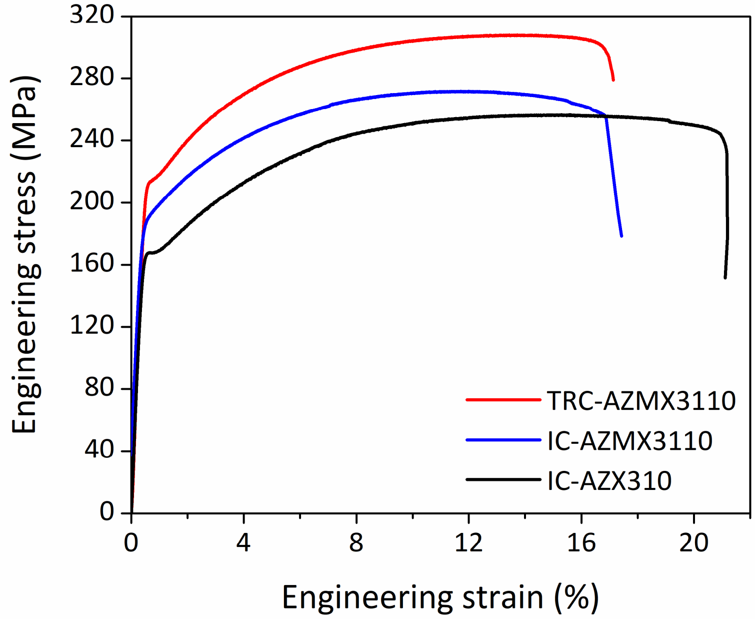

Supplementary Figure 1: Tensile stress-strain curves of the studied alloys

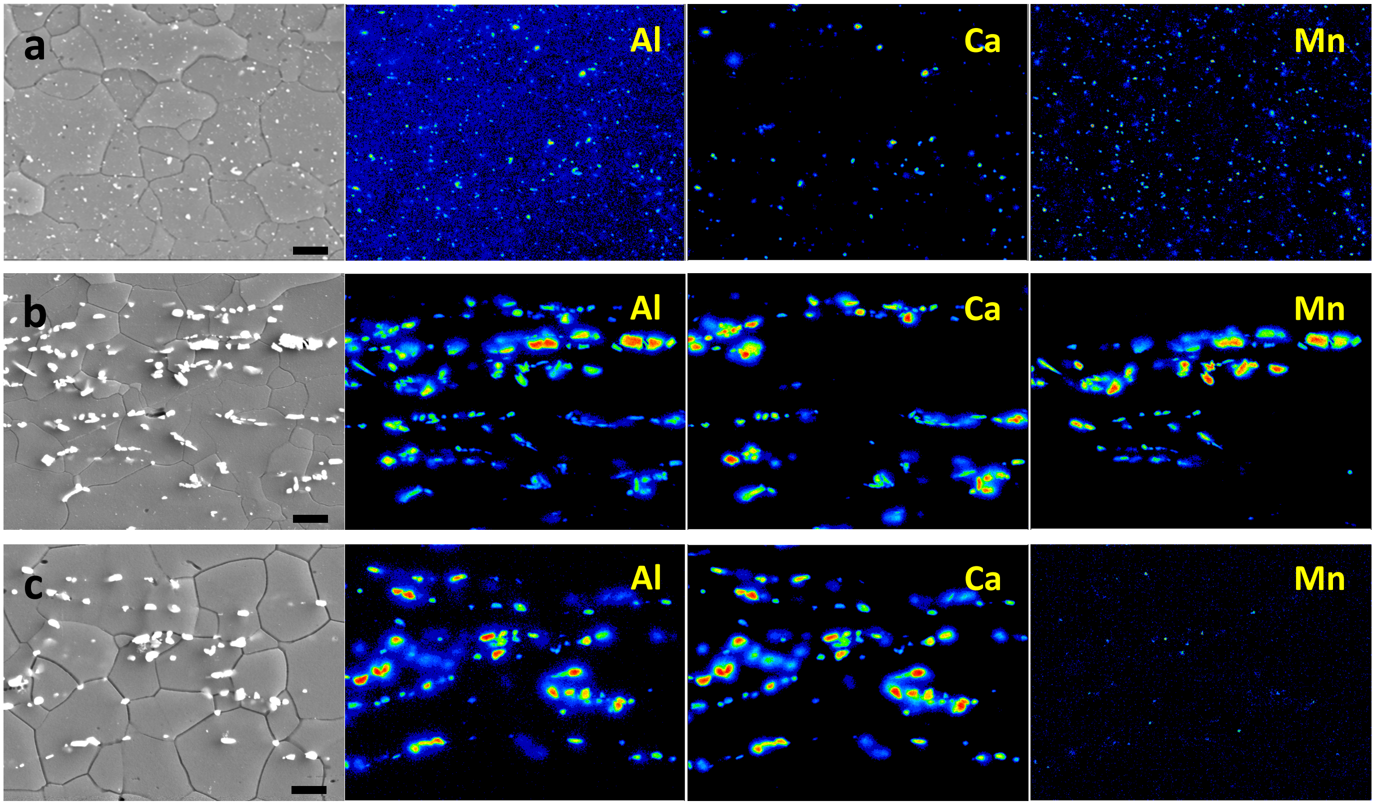

Supplementary Figure 2: Identification of coarse second phase particles by EPMA. EPMA elemental mapping images showing the compositions of coarse second phase particles in TRC-AZMX3110 (**a**), IC-AZMX3110 (**b**) and IC-AZX310 (**c**). The coarse second phase particles contain Al and Ca (Al_2Ca type) and/or Al and Mn (Al_8Mn_5 type). While all the particles in IC-AZX310 are Al_2Ca, IC- and TRC-AZMX3110 contain both Al_2Ca and Al_8Mn_5 particles. Scale bar corresponds to 5 μm.

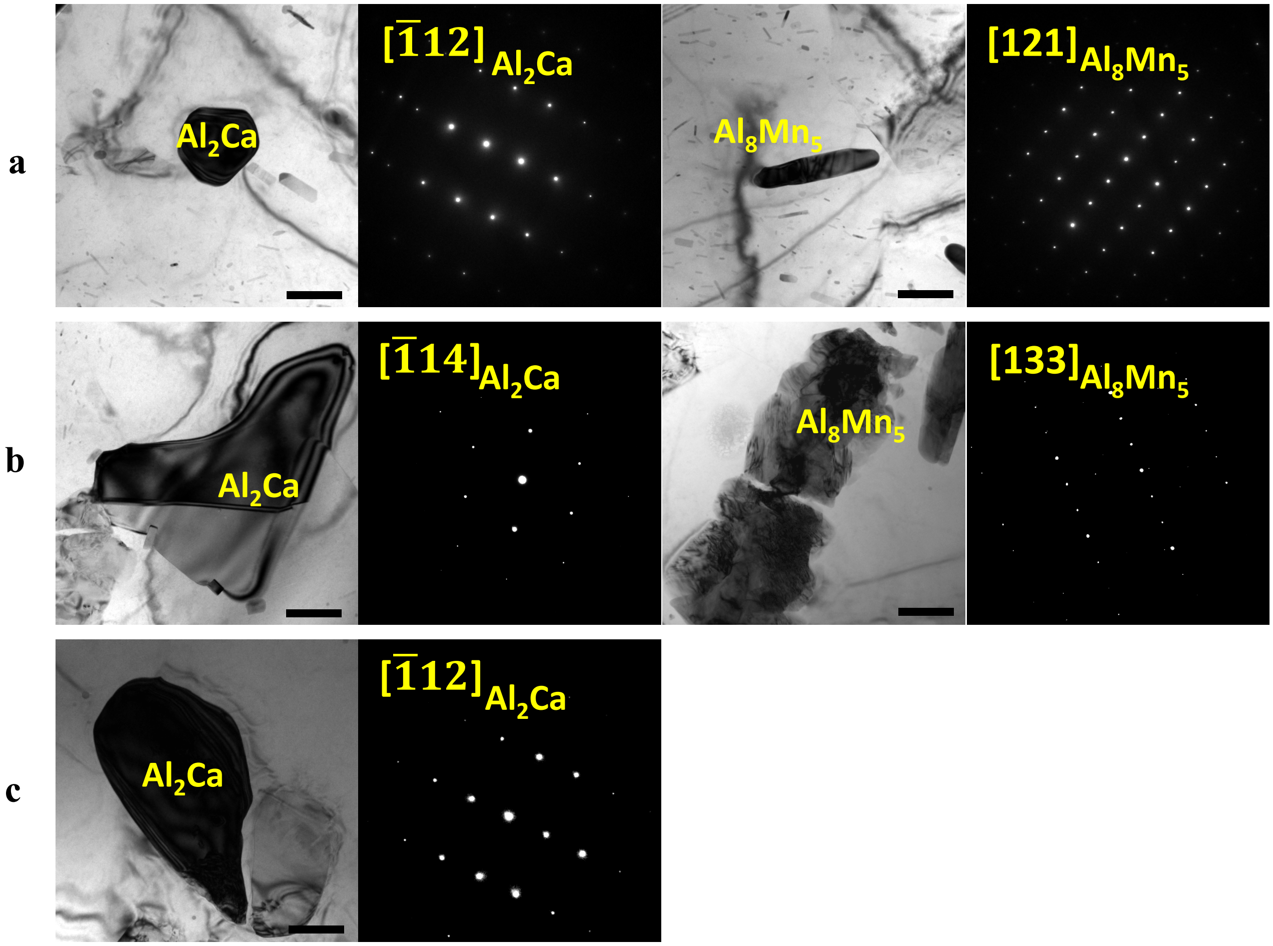

Supplementary Figure 3: Identification of coarse second phase particles by TEM. TEM-BF and corresponding TEM-DPs of coarse second phase particles identified as Al_2Ca particles having FCC structure (a = 0.807 nm) and Al_8Mn_5 particles having a rhombohedral structure (a = b = c = 0.78 nm, $\alpha = \beta = \gamma = 109.21^{o}$) in: (**a**) TRC-AZMX3110, (**b**) IC-AZMX3110, and (**c**) IC-AZX310. Scale bar corresponds to 500 nm.

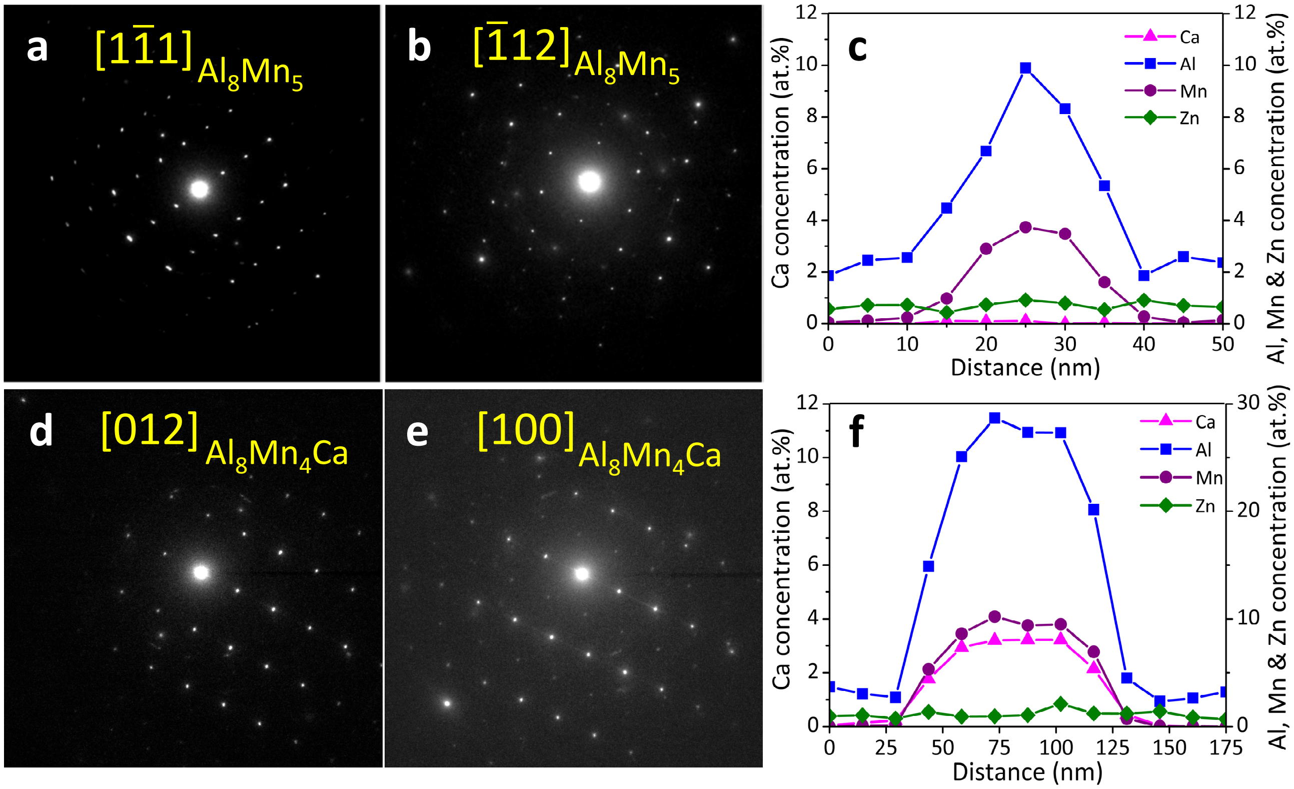

Supplementary Figure 4: Identification of fine plate-shaped particles in TRC-AZMX3110 by TEM-DPs and EDS analyses. **a, b**, TEM-DPs from two different zone axes of a Al_8Mn_5 particle having a rhombohedral structure (a = b = c = 0.78 nm, α = β = γ = 109.21°): [1$\bar{1}$1] (**a**) and [$\bar{1}$12] (**b**) zone axes (using trigonal setting). **c**, TEM-EDS spectra showing the composition of a Al_8Mn_5 particle. **d, e**, TEM-DPs from [012] (**d**) and [100] (**e**) zone axes of a Al_8Mn_4Ca particle having a tetragonal structure (a = 0.895 nm, c = 0.516 nm). **f**, EDS spectra showing the composition of a Al_8Mn_4Ca particle.

Supplementary Table 1: Analyzed chemical compositions of the studied alloys

Alloys		Mg	Al	Zn	Mn	Ca
**TRC - AZMX3110**	wt.%	94.79	2.83	1.05	0.88	0.45
	at.%	96.34	2.59	0.4	0.4	0.28
**IC - AZMX3110**	wt.%	94.61	2.93	1.06	0.82	0.58
	at.%	96.19	2.68	0.4	0.37	0.36
**IC - AZX310**	wt.%	95.86	2.70	0.97	-	0.47
	at.%	96.89	2.46	0.36	-	0.29

Supplementary Table 2: Summary of tensile properties and formability of the studied alloys

Alloys	YS (MPa)	UTS (MPa)	El. (%)	***n***	IE (mm)
**TRC-AZMX3110**	219	306	17	0.197	8.0
**IC-AZMX3110**	190	272	17	0.188	5.0
**IC-AZX310**	167	256	21	0.242	4.8

YS: yield strength, UTS: ultimate tensile strength, El.: total elongation,
n: strain hardening exponent, and I.E.: index Erichsen value.
